# Prevalence and molecular characterization of *Cryptosporidium* spp. and *Giardia duodenalis* in dairy cattle in Ningxia, northwestern China

**DOI:** 10.1186/s12917-014-0292-6

**Published:** 2014-12-09

**Authors:** Jianying Huang, Daoyou Yue, Meng Qi, Rongjun Wang, Jinfeng Zhao, Junqiang Li, Ke Shi, Ming Wang, Longxian Zhang

**Affiliations:** College of Animal Science and Veterinary Medicine, Henan Agricultural University, Zhengzhou, 450002 China; International Joint Research Laboratory for Zoonotic Diseases of Henan, Zhengzhou, 450002 China; College of Veterinary Medicine, China Agricultural University, Beijing, 100193 China

**Keywords:** *Cryptosporidium*, *Giardia duodenalis*, Dairy cattle, SSU rRNA, gp60, tpi, gdh

## Abstract

**Background:**

*Cryptosporidium* spp. and *Giardia duodenalis* are important gastrointestinal protists in humans and animals worldwide. In China, bovine cryptosporidiosis and giardiasis are of increasing concern because cattle are important reservoirs of these parasites, which have become potential threats to public health and to large numbers of cattle in recent years.

**Results:**

A total of 1366 fecal samples from the Ningxia Autonomous Region were examined. The overall infection rates for *Cryptosporidium* spp. and *G. duodenalis* were 1.61% and 2.12%, respectively. *Cryptosporidium* was only detected in preweaned calves and adults older than 2 years, whereas *G. duodenalis* was only detected in calves aged less than 11 months. *Cryptosporidium* spp. were characterized with a PCR–restriction fragment length polymorphism analysis and DNA sequence analysis of the small subunit rRNA gene. Three *Cryptosporidium* species were identified: *C. parvum* (n = 15) and *C. bovis* (n = 4) in preweaned calves, and *C. andersoni* (n = 4) in adults aged over 2 years. A DNA sequence analysis of the gp60 gene suggested that the 15 *C. parvum* isolates all belonged to subtype IIdA15G1. Twenty-nine *G. duodenalis* isolates were analyzed by DNA sequencing of the triosephosphate isomerase (tpi) and glutamate dehydrogenase (gdh) genes. Two *G. duodenalis* assemblages were identified, assemblages E (n = 15) and B (n = 4, one subtype B1 and three subtype B2) in preweaned calves, and assemblage E (n = 10) in 3–11-month-old calves.

**Conclusions:**

The predominance of *C. parvum* detected in preweaned calves and the first identified subtype IIdA15G1 in dairy cattle, and the dominant *G. duodenalis* assemblage E in this study differed considerably from those found in Henan, Heilongjiang, and Shannxi Provinces. Our findings further confirm the dominance of *C. parvum* IId subtypes in China.

## Background

*Cryptosporidium* and *Giardia* are important gastrointestinal protists with a wide spectrum of hosts, including humans, livestock, companion animals, and wildlife. Infection is acquired via the fecal–oral route following the ingestion of infective oocysts or cysts, by either direct contact or the ingestion of contaminated food or water [1,2]. Diarrhea is the typical clinical symptom of human and animal cryptosporidiosis or giardiasis, as well as dehydration, fever, nausea, and anorexia.

Cattle are mammalian species commonly infected with *Cryptosporidium*, and preweaned calves are considered the most important reservoir for zoonotic infections. Large numbers of studies have suggested that *C. parvum*, *C. bovis*, *C. andersoni*, and *C. ryanae* are the most common species infecting cattle, although *C. felis*, *C. hominis*, *C. suis*, *C. scrofarum* (formerly pig genotype II), and *C. suis*-like genotype have also been detected [3]. The four common *Cryptosporidium* species have age-associated distributions. *Cryptosporidium parvum* is usually found in preweaned calves and is a significant cause of diarrhea. However, *C. bovis* and *C. ryanae* usually infect postweaned calves and yearlings, and *C. bovis* is detected more frequently than *C. ryanae*, although neither is associated with diarrhea [4]. In contrast, *C. andersoni* is commonly seen in adult cattle and is associated with gastritis, reduced milk yield, and poor weight gain [5].

Among the six known *Giardia* species, only *G. duodenalis* is recovered from humans and most other mammals [6]. Its molecular characterization has shown that *G. duodenalis* is a species complex comprising eight distinct ‘assemblages’ or genotypes: A to H [2]. Most of the assemblages (C to H) seem to be host specific for nonhuman species: assemblages C and D are specific for dogs, E for hoofed livestock, F for cats, G for rats, and H for seals. In cattle, the prevalence of giardiasis ranges from 2.2% to 50.7% worldwide, and assemblages A, B, and E have been detected, with assemblage E the predominant genotype in most countries [1,2].

In China, bovine cryptosporidiosis and giardiasis are of increasing concern because cattle are important reservoirs of these parasites, which have become potential threats to public health and to large numbers of cattle in recent years. Several studies of *Cryptosporidium* and *Giardia* infections in several areas have been published in the English language [7-11], and differences in *Cryptosporidium* distributions have been noted in different locations. For example, *C. bovis* rather than *C. parvum* is the predominant species in preweaned calves in Henan, whereas *C. andersoni* is the most common species of cattle in Heilongjiang and Shannxi Provinces [7,12]. Thus, the distributions of *Cryptosporidium* spp. and the *C. parvum* subtype (all belonging to IIdA19G1) in dairy cattle differ considerably from those in other countries [9,10]. In contrast, there have been few molecular epidemiological studies of *G. duodenalis* in cattle [10,13]. The objective of this study was to identify the species of *Cryptosporidium* and *Giardia* present in dairy cattle in the Ningxia Autonomous Region, northwest China.

## Methods

### Ethics statement

This study was conducted in accordance with the Chinese Laboratory Animal Administration Act of 1988. The research protocol was reviewed and approved by the Research Ethics Committee of the Henan Agricultural University. Permission was obtained from farm owners before collection of fecal samples.

### Sample collection and examination

A fresh fecal sample was collected from each animal using a sterile disposal latex glove immediately after its defecation onto the ground, and was then placed individually into a disposable plastic bag. In total, 1366 fecal samples were collected between December 2011 and October 2012 from dairy cattle on 20 scale farms in the Ningxia Autonomous Region, northwestern China (Table 1). The *Cryptosporidium* oocysts in the fecal materials were concentrated with Sheather’s sugar flotation technique, with a further formalin–ethyl acetate sedimentation step included for the preweaned calf samples [9]. *Giardia* cysts were detected with Lugol’s iodine staining. *Cryptosporidium*- or *Giardia*-positive samples were stored in 2.5% potassium dichromate at 4°C before DNA extraction.

**Table 1:** **Numbers of fecal samples examined with microscopy for** ***Cryptosporidium*** **oocysts and** ***Giardia*** **cysts on Chinese farms**

**Farm**	**Sample size**	**Prevalence (%)** ***Cryptosporidium*** **/** ***Giardia***	***Cryptosporidium*** **species (no.)**	***Giardia duodenalis*** **assemblage (no.)**
1	45	2.22%/2.22%	*C. bovis* (1)*	B2 (1)*
2	32	9.38%/6.25%	*C. parvum* (1)**, *C. bovis* (2)**	B2 (2)*
3	57	3.51%/3.51%	*C. parvum* (2)***	E (1), B1 (1)***
4	46	4.35%/6.52%	*C. parvum* (2)	E (3)
5	63	3.17%/7.94%	*C. parvum* (2)	E (5)
6	106	0.94%/1.89%	*C. parvum* (1)	E (2)
7	110	6.36%/3.64%	*C. parvum* (7)	E (4)
8	166	0/1.20%		E (2)
9	94	4.26%/3.19%	*C. andersoni* (4)	E (3)
10	32	0/6.25%		E (2)
11	141	0/1.42%		E (2)
12	76	0/1.32%		E (1)
13	69	1.45%/0	*C. bovis* (1)	
14-20	329	0/0		
Total	1366	1.68%/2.12%	*C. parvum* (15), *C. bovis* (4), *C. andersoni* (4)	

The distributions of *Cryptosporidium* spp. and *G. duodenalis* assemblages were determined with PCR–RFLP analysis of the SSU rRNA gene and sequence analysis of the *tpi* and *gdh* genes.

*Sample positive for a mixed infection of *C. parvum* and *G. duodenalis* subtype B2.

**One sample showed a mixed infection of *C. parvum* and *G. duodenalis* subtype B2, and another sample showed a mixed infection of *C. bovis* and *G. duodenalis* subtype B2.

***Sample positive for a mixed infection of *C. parvum* and *G. duodenalis* subtype B1.

### DNA extraction

The *Cryptosporidium* or *Giardia*-positive fecal specimens were washed three times with distilled water, and the genomic DNA was extracted from the fecal pellets with the E.Z.N.A.® Stool DNA Kit (Omega Biotek Inc., Norcross, GA, USA), according to the manufacturer’s recommendations.

### *Cryptosporidium/Giardia* genotyping and subtyping

The *Cryptosporidium* species were identified with PCR–restriction fragment length polymorphism (RFLP) analysis and DNA sequence analysis of the small subunit (SSU) rRNA gene [14]. *Cryptosporidium parvum* was subtyped with nested PCR targeting the gp60 gene, and the previously established nomenclature system was used to name the *C. parvum* subtype families and subtypes [15,16]. The *Giardia duodenalis* genotypes/subtypes were identified by sequencing the triosephosphate isomerase (tpi) and glutamate dehydrogenase (gdh) genes [17], and the genotype/subtype identities of the *G. duodenalis* samples were established by direct comparison of these sequences with reference sequences downloaded from the GenBank database.

### DNA sequence analysis

PCR products were sequenced on an ABI Prism™ 3730 XL DNA Analyzer using the BigDye Terminator v3.1 Cycle Sequencing Kit (Applied Biosystems, Foster, CA, USA). Sequence accuracy was confirmed with bi-directional sequencing and by sequencing a new PCR product if necessary. The sequences were aligned with the ClustalX 1.83 program. Representative nucleotide sequences have been deposited in GenBank under accession numbers KM067089–KM067096.

### Statistical analysis

The χ^2^ test was used to compare the *Cryptosporidium* infection rates. Differences were considered significant at P <0.05.

## Results

### Prevalence of *Cryptosporidium* and *Giardia*

Microscopic analysis of 1366 fecal samples showed the presence of *Cryptosporidium* oocysts in 23 samples (1.68%) from nine farms, and the highest infection rate was 9.38% on farm 2 (Table 1). The infection rates of *Cryptosporidium* spp. were 10.22%, 0%, 0%, and 0.69% in preweaned, 3–11-month-old, 1–2-year-old, and > 2-year-old cattle, respectively (χ^2^ = 95.52, P <0.01).

Similarly, 29 samples from 12 farms were positive for *Giardia*, with an average infection rate of 2.12% (Table 1). The highest infection rate for *Giardia* was 7.94% on farm 5. The infection rates for *Giardia* were 10.22%, 2.53%, 0%, and 0% in preweaned, 3–11-month-old, 1–2-year-old, and > 2-year-old cattle, respectively (χ^2^ = 75.93, P <0.01).

### Distribution of *Cryptosporidium* species/subtypes and *G. duodenalis* assemblages

The SSU rRNA genes of *Cryptosporidium* spp. in all 23 microscopy-positive samples were successfully amplified with nested PCR. RFLP and DNA sequence analyses of the SSU rRNA gene fragments revealed the presence of three *Cryptosporidium* species: *C. parvum* (n = 15) on six farms, *C. bovis* (n = 4) on three farms, and *C. andersoni* (n = 4) on one farm (Table 1). With the exception of farm 2, only one *Cryptosporidium* species was detected on all the *Cryptosporidium*-positive farms (Table 1). The gp60 sequencing analysis showed that the 15 *C. parvum* isolates all belonged to subtype IIdA15G1.

Sequencing analyses of the tpi and gdh genes in *G. duodenalis* identified two assemblages: E (n = 25) on 10 farms and B (n = 4) on three farms (Table 1). Only farm 3 had both assemblages E and B.

### Age distributions of *Cryptosporidium* and *G. duodenalis*

*Cryptosporidium parvum* was the most commonly identified *Cryptosporidium* species, responsible for 65.2% of all *Cryptosporidium* infections, and was only found in preweaned calves (Table 2). *Cryptosporidium bovis* and *C. andersoni* were found in preweaned calves and > 2-year-old cattle, respectively. In contrast, no *Cryptosporidium*-positive sample was identified in cattle aged between 3 months and 2 years.

**Table 2:** ***Cryptosporidium*** **species identified in dairy cattle in different age groups**

**Age group**	**Sample size**	***Cryptosporidium***		***G. duodenalis*** **assemblage**	
		**Prevalence**	**Species (no.)**	**Prevalence**	**Assemblage (no.)**
Preweaned*	186	10.22%	*C. parvum* (15), *C. bovis* (4)	10.22%	E (15), B1 (1), B2 (3)
3–11 months	396	0	0	2.53%	E (10)
1–2 year	204	0	0	0	0
> 2 years	580	0.69%	*C. andersoni* (4)	0	0

*Among the positive samples from preweaned calves, one was a mixed infection of *C. bovis* and *G. duodenalis* subtype B2, one was a mixed infection of *C. parvum* and *G. duodenalis* subtype B1, and two were mixed infections of *C. parvum* and *G. duodenalis* subtype B2.

*Giardia duodenalis* was only detected in preweaned and 3–11-month-old calves. Among these calves, *G. duodenalis* assemblage E was the dominant assemblage, responsible for 86.2% (25/29) of all *Giardia*-positive samples. In contrast, only four assemblage B infections (one subtype B1 and three subtype B2) were found in preweaned calves. Four mixed infections of *Cryptosporidium* and *G. duodenalis* were found in preweaned calves (Table 2).

## Discussion

The overall infection rate for *Cryptosporidium* spp. was 1.68%, which is lower than those in Henan (13.0%, 276/2116) [8,9], Heilongjiang (15.0%, 99/658) [7,11], Shannxi (3.4%, 70/2071) [12], Anhui (14.9%, 52/350), Jiangsu (20.7%, 251/1215), and Shanghai (12.5%, 55/440) [18]. The average infection rate for *G. duodenalis* was 2.12%, which is lower than those in Heilongjiang (5.2%, 42/814) [10] and Henan (7.2%, 128/1777) [13]. Oocysts shed at low intensity may be contributed to the low detecting rates of both parasites by microscopy. In general, it is difficult to explain the actual discrepancies in the prevalence of *Cryptosporidium* spp. and *G. duodenalis* among different studies because the infection rates are related to many factors, including the examination methods, age distributions of the animals, sample sizes, host health status at the time of sampling, the timing of specimen collection, and geo-ecological conditions. Nevertheless, the infection rates of both parasites were always higher in preweaned calves than in any other age group, which is consistent with previous observations [8-10,19,20].

Among the preweaned calves, *C. parvum* was the predominant *Cryptosporidium* species, rather than *C. bovis*. This result clearly differs from the results reported in Henan and Heilongjiang, where *C. bovis* was the dominant species, and in Shannxi, where only *C. andersoni* was detected [9,11,12]. The results of most previous studies, conducted in numerous countries, suggest that *C. parvum* is the predominant *Cryptosporidium* species in preweaned calves [9]. In contrast, only *C. andersoni* was detected in adult cattle in the present study, which was basically similar to the reports of *Cryptosporidium* infections in cattle in Henan, Heilongjiang, Shannxi, and other countries [7,8,12,21].

The 15 *C. parvum* isolates were identified as subtype IIdA15G1 based on a sequence analysis of the gp60 gene, which differed from subtype IIdA19G1 found in dairy cattle in Henan and Heilongjiang provinces, China [9,11]. In fact, except for *C. parvum* IIa subtypes found in yaks [22], the *C. parvum* isolates characterized in China thus far have belonged to the IId subtypes, including IIdA15G1 in rodents [23], IIdA19G1 in cattle, humans, and urban wastewater [9,11,24,25]. Thus, the characteristics of *C. parvum* subtypes appear to be unique in China. Sequence analysis of the gp60 gene has been used extensively to characterize the molecular epidemiology of cryptosporidiosis in animals and humans, and at least 14 *C. parvum* subtype families have been identified: IIa, IIb, IIc, IId, IIe, IIf, IIg, IIh, IIi, IIk, IIl, IIm, IIn, and IIo [1,16]. Of these, IIa is the predominant subtype family in animals and humans worldwide, and IId is another major zoonotic subtype family reported in Europe (Hungary, Germany, Portugal, Sweden, Ireland, Spain, Belgium, Romania, United Kingdom, Netherlands, Slovenia, Serbia, and Montenegro), Asia (Kuwait, Iran, Jordan, India, Malaysia, and China), Egypt, and Australia [9,26-29]. In contrast, IIc and IIe are anthroponotic subtype families. Other subtype families of *C. parvum* are occasionally -seen in various animals or humans around the world [9,16].

Sequence analyses of the tpi and gdh genes showed that most of the dairy cattle were infected with livestock-specific *G. duodenalis* assemblage E (86.2%). This result is consistent with a previous study conducted in Heilongjiang and Henan, China [10,13]. The results of most studies conducted in numerous countries, including Belgium, Denmark, Portugal, Spain, Sweden, Germany, UK, USA, Canada, Brazil, Australia, Rwanda, Egypt, Sri Lanka, and Malaysia, suggest that assemblage E is the predominant *Giardia* assemblage in cattle [2,30-39].

*Giardia duodenalis* assemblage B was only found in four preweaned calves in this study. In a previous study conducted in Heilongjiang Province, a higher rate of assemblage B was identified (18/43) in dairy cattle [10], whereas no assemblage B was found in Henan Province [13]. In general, assemblage B has been detected in only a small number of cattle in a few studies worldwide, including in Europe, Italy, Portugal, Uganda, New Zealand, and Canada [2,30]. Assemblage B, one of the two major assemblages causing human giardiasis, has a broad host range, including cattle, sheep, pigs, horses, dogs, cats, and rabbits [2]. Although no strong evidence supporting the direct zoonotic transmission of *G. duodenalis* from animals to humans has been reported, case–control studies have shown that contact with farm animals increases the infection rate of human giardiasis [40,41]. Considering how large the cattle industry is and the close contact that occurs between cattle and humans, assemblage B *G. duodenalis* identified in cattle may emerge as an important zoonotic pathogen in some areas of China.

## Conclusion

In summary, *C. parvum* is the predominant species in preweaned calves in the study area, which clearly differs from the situation in Henan, Heilongjiang, and Shannxi Provinces. The presence of *C. parvum* subtype IIdA15G1, together with subtypes IIdA15G1 and IIdA19G1, previously found in rodents, ruminants, and humans, further confirms the dominance of the *C. parvum* IId subtypes in China. The dominant *G. duodenalis* assemblage, assemblage E, is similar to the dominant assemblages in other areas or countries.
